# Tuberculosis screening and follow-up of asylum seekers in Norway: a cohort study

**DOI:** 10.1186/1471-2458-9-141

**Published:** 2009-05-14

**Authors:** Ingunn Harstad, Einar Heldal, Sigurd L Steinshamn, Helge Garåsen, Geir W Jacobsen

**Affiliations:** 1 Department of Public Health and General Practice, Norwegian University of Science and Technology, Trondheim, Norway; 2 Norwegian Institute of Public Health, Oslo, Norway; 3 Department of Circulation and Medical Imaging, Norwegian University of Science and Technology, Trondheim, Norway; 4 Department of Pulmonary Medicine, St. Olavs University Hospital, Trondheim, Norway; 5 City of Trondheim, Department of Health and Social Welfare, Trondheim, Norway

## Abstract

**Background:**

About 80% of new tuberculosis cases in Norway occur among immigrants from high incidence countries. On arrival to the country all asylum seekers are screened with Mantoux test and chest x-ray aimed to identify cases of active tuberculosis and, in the case of latent tuberculosis, to offer follow-up or prophylactic treatment.

We assessed a national programme for screening, treatment and follow-up of tuberculosis infection and disease in a cohort of asylum seekers.

**Methods:**

Asylum seekers ≥ 18 years who arrived at the National Reception Centre from January 2005 to June 2006, were included as the total cohort. Those with a Mantoux test ≥ 6 mm or positive x-ray findings were included in a study group for follow-up.

Data were collected from public health authorities in the municipality to where the asylum seekers had moved, and from hospital based internists in case they had been referred to specialist care.

Individual subjects included in the study group were matched with the Norwegian National Tuberculosis Register which receive reports of everybody diagnosed with active tuberculosis, or who had started treatment for latent tuberculosis.

**Results:**

The total cohort included 4643 adult asylum seekers and 97.5% had a valid Mantoux test. At least one inclusion criterion was fulfilled by 2237 persons. By end 2007 municipal public health authorities had assessed 758 (34%) of them. Altogether 328 persons had been seen by an internist. Of 314 individuals with positive x-rays, 194 (62%) had seen an internist, while 86 of 568 with Mantoux ≥ 15, but negative x-rays (16%) were also seen by an internist. By December 31^st ^2006, 23 patients were diagnosed with tuberculosis (prevalence 1028/100 000) and another 11 were treated for latent infection.

**Conclusion:**

The coverage of screening was satisfactory, but fewer subjects than could have been expected from the national guidelines were followed up in the community and referred to an internist. To improve follow-up of screening results, a simplification of organisation and guidelines, introduction of quality assurance systems, and better coordination between authorities and between different levels of health care are all required.

## Background

As tuberculosis (TB) in native populations in Western countries decreases, the relative importance of cases among immigrants increases. Latent tuberculosis is prevalent in immigrants, and may result in an increased incidence for many years after immigration [1,2].

Many Western countries carry out screening immediately after arrival, but programmes for immigrants from high incidence countries vary widely between them, and their documented impact is sparse. While some countries focus mainly on diagnosing active pulmonary tuberculosis, others follow up high risk individuals, or aim at preventing new cases through BCG immunisation or treatment of latent tuberculosis [3,4].

For public health it is most important to identify cases of pulmonary tuberculosis and the majority of them can be detected by chest x-ray [1,5]. Still many cases of extra-pulmonary tuberculosis and latent tuberculosis may thus be overlooked. A tuberculin skin test is often used in addition to x-ray, but has several limitations [6]. Recently, new interferon gamma release assays have been introduced, with promising results for diagnosing latent tuberculosis and may become a useful addition to the screening programme [7].

In Norway, the incidence of tuberculosis in the general population gradually decreased until the late 1980's. Thereafter the number of new cases has remained low and was 6.3/100 000 population in 2006 [8]. During the last 25 years the characteristics of incident cases have changed, and the active disease now stem from imported strains rather than person to person transmission within the country [9]. Somalia is the country of origin for most new cases.

Previously, the main focus of the Norwegian tuberculosis control programme was on early case detection of active disease and follow-up without treatment of the latent form. In 2002 the European working group on tuberculosis control and elimination in low incidence countries (WHO, International Union Against Tuberculosis and Lung Disease, Royal Netherlands Tuberculosis Association) recommended a new strategy. It aimed to reduce the prevalence of tuberculosis infection and also included prophylactic therapy [10].

The same year new regulations for tuberculosis control were introduced in Norway. These emphasised a continued need to screen immigrants from high incidence countries after arrival, as well as others with high risk, and to follow up those with abnormal findings [11]. The corresponding national guidelines, that were issued by the national health authorities to all health personnel engaged in TB management and care, promoted more vigorous treatment of latent tuberculosis [12].

Public health care in Norway is organised in two levels where the municipal authorities are responsible for primary health care. This includes infectious disease control within the community. Patients in whom screening outcome implies a more detailed and targeted follow-up, are referred to specialist care in state-owned hospitals.

### Recommended screening and management of tuberculosis

**Figure 1:**
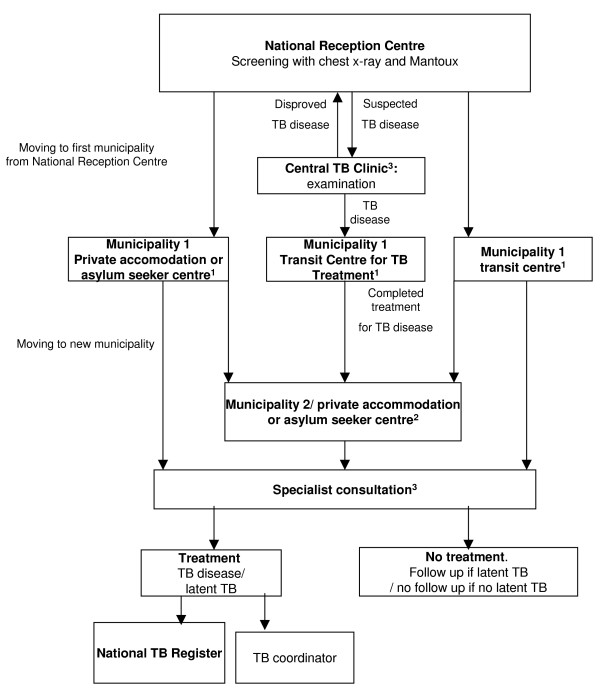
**Flow of asylum seekers**. Primary Health Care (PHC) form sent to first municipality (transit centres, asylum seeker centres, or private accommodation) 2: PHC form sent to second municipality (asylum seeker centres or private accommodation) 3: Specialist form sent to the Central TB Clinic and to other hospital based specialists (internists).

Asylum seekers and refugees (table 1) are subgroups of immigrants with particularly high risk for tuberculosis [13,14]. Hence, on arrival in Norway, all asylum seekers are referred to the National Reception Centre outside Oslo for registration, management of immediate medical needs, and compulsory tuberculosis screening (figure 1 and table 2). Screening includes a Mantoux test and chest x-ray for everyone over 15 years of age.

**Table 1:** Definitions of "asylum seeker" and "refugee"

Asylum seeker^1^	A person who on his or her own initiative, and without prior notification, asks the authorities in Norway for protection and recognition as a refugee. The person is called an asylum seeker until a decision has been made on the application.
	
Refugee	A person who either has been granted residence permit before arrival, through the UN system, or arrived as an asylum seeker and has been granted protection as refugee after application.

^1^

**Table 2:** Kind and characteristics of institutions attending asylum seekers

**Institutions (total number)**	**Functions**	**Health care level**	**Staff**	**Source of information for the study**
**National Reception Centre (1)^1^**	Short term. All asylum seekers (AS) on arrival to Norway. Screening by chest x-ray and Mantoux.	Primary health care (PHC) paid for by national government.	PHC officer or nurse	Information from the computer system of the National Reception Centre.
**TB Treatment Transit Centre (1)^1^**	Short or long term. Treatment of patients with active TB, or potential patients waiting for consultation or examination results. Family members are included.	PHC paid for by national government	PHC officer or nurse	Filled in PHC registration forms^2^
**Central TB Clinic (1)^1^**	Out patient. AS referred directly from the National Reception Centre or from other levels. Focus is active TB.	Hospital based specialist health care.	Specialists in pulmonary medicine	Filled in specialist registration form^3^
**Transit centre (4)^1^**	Short term stay. AS who wait for an interview by police or for being deported.	PHC paid for by national government	PHC officer or nurse	Filled in PHC registration forms^2^
**Asylum seeker centre (ca 100, January 2005)**	Longer stay. Staying there till they get a permanent residency or are deported.	Municipal authorities directly responsible for health care.	PHC officer or nurse	Filled in PHC registration forms^2 ^or ^4^
**Specialist health care**	Out patient. Examine and treat AS after referral from primary health care	Hospital based specialist care.	Hospital specialists, pulmonary or internal medicine	Filled in specialist registration form^3^

^1^: Number of centres, ^2^: PHC forms sent to the first municipality after the National Reception Centre, ^3^: Specialist forms, ^4^: PHC forms sent to the second municipality after the National Reception Centre

According to the guidelines, asylum seekers with a chest x-ray suggestive of tuberculosis disease, as well as everyone who reports symptoms of tuberculosis, should be referred directly to the Central TB Clinic at Ullevaal University Hospital, Oslo, for further examination. It is clearly stated that in addition to a positive x-ray, a Mantoux test ≥ 15 mm should lead to a direct referral to an internist [12]. When leaving the National Reception Centre, asylum seekers are directed to one of several local asylum seeker centres or transit centres in communities throughout the country. If an individual has to await the outcome of examination, or if treatment is started, the whole family will stay at one specially designated Transit Centre for TB Treatment until the treatment is completed (figure 1). Those who initially stay in a transit centre later move on to an asylum seeker centre. On the other hand, they may also leave the country on their own or be sent out at any time. Sometimes, asylum seekers find private accommodation on their own with help from family or others.

Screening results are mailed to the respective municipal health authorities, and everyone with a Mantoux test of 6–14 mm should be clinically examined in primary health care and interviewed about other TB risk factors [12]. An HIV test is not compulsory, but is taken if deemed clinically relevant. Whenever definite risk factors are identified, all patients should be referred to an internist who is responsible for ensuring timely clinical examination and a sputum test, and any other relevant diagnostic work-up. If tuberculosis disease is diagnosed or treatment for latent tuberculosis is started, a nominal notification is sent to the National Tuberculosis Register.

Information about asylum seekers is readily available since they are all received through a single National Reception Centre.

We aimed to assess to what extent the national recommendations for screening, treatment and follow-up of tuberculosis disease and infection among asylum seekers had been implemented at the primary and specialist health care levels in Norway [11,12].

## Methods

### Study population

All asylum seekers ≥ 18 years who arrived at the National Reception Centre from January 2005 to June 2006, were eligible for study inclusion except those that had left the centre without a forwarding address, had left the country, had been deported, or had died before leaving the centre.

Inclusion into the study group for follow-up was ascertained from information held in the data base at the National Reception Centre. The criteria for inclusion were either a positive Mantoux test or a positive chest x-ray. The former was defined as Mantoux ≥ 6 mm, (PPD: RT 23, 2 TU from SSI, Copenhagen, Denmark).

X-ray films were taken at the National Reception Centre and interpreted by two independent readers, a pulmonologist and a radiologist. The results were later recoded for this study. A positive x-ray included pleural pathology, pulmonary, hilar or mediastinal calcifications, or parenchymal pathology.

An interferon gamma release assay test (QuantiFERON^® ^TB Gold in-tube test, Cellestis Ltd, Carnegie, Victoria, Australia, QFT) had been performed in a previous study of a subset of 912 eligible persons [15].

### Data collection

Information on demographics and initial screening results was collected from the data base at the National Reception Centre. Follow-up information was obtained by using two different study forms. First, a local primary health care (PHC) form was sent to the public health physician in the municipality where the asylum seekers had moved to. If the person in question had moved to a second municipality, the same form was sent there (figure 1).

Second, a specialist form was sent to the Central TB Clinic whenever findings at the National Reception Centre had indicated that a referral was indicated. It was sent to other hospital internists throughout the country when the PHC form positively confirmed that an individual had been referred to specialist care (figure 1).

Both forms collected information about demographics, registration and flow of information, previous history and other diseases, TB risk factors, symptoms, clinical examination and findings, plans for follow-up or referrals, and relocations of study participants. The municipalities were asked whether the asylum seeker had been examined or interviewed, and if so, by whom: physician, community health nurse, or regular nurse. Any referrals were recorded: x-rays, specialist health care, or others. In case of no referral, the reason why was recorded: not indicated, the patient did not want a referral, or other reasons. Relevant dates and numbers were also recorded.

Name and birth date on everyone included in the study group were checked against the National Tuberculosis Register. The latest update of the register took place on December 31^st ^2006.

### Data handling and analysis

Study forms were scanned and entered into SPSS for Windows, version 14 (Chicago, IL, USA). Comments and administrative information were coded manually and entered to the same data file.

Frequencies were analyzed with proportions and 95% confidence intervals (CI). Groups were compared on demographics, and positive x-rays were also compared on x-ray codes. Prevalence < 0.05 were considered statistically significant.

### Study ethics

The Regional Committee for Medical Research Ethics approved the study. The Norwegian Data Inspectorate, the Directorate for Health and Social Affairs, the Ministry of Labour and Social Inclusion, and the Research Committee at Ullevaal University Hospital all gave their permission.

## Results

Among the 5112 asylum seekers ≥ 18 years who arrived at the National Reception Centre from January 2005 to June 2006, 4643 (91%) were eligible for study inclusion and are hereafter referred to as the total cohort. Among this cohort, there were 3222 (69%) males and 3333 (72%) were in the 18–34 year old age group. They came from 90 different countries, and Iraq, Somalia, Russia, Afghanistan, and Serbia and Montenegro contributed most, i.e. 2434 persons (52%). Coverage of a valid Mantoux test was 4526 (97.5%), the yield of a Mantoux ≥ 6 mm was 2127 (46%) and a positive chest x-ray 323 (7%) individuals, respectively. A positive QFT test was used as the only inclusion criterion for 28 participants.

### The follow-up study group

Of the 2293 asylum seekers with at least one positive inclusion criterion, 2237 were included in the follow-up study because 56 individuals who were eligible for inclusion had left the National Reception Centre with no forwarding address, left the country, been deported, or died before leaving the centre.

The proportion of males was higher in the youngest age group. More participants came from Europe and Asia in the older age group while there were more Africans in the youngest one. The five most frequent countries of origin were the same as in the total cohort.

Registration forms to local municipalities were sent to 81 different asylum seekers centres. From January 2005 to January 2007 there was a reduction in the number of asylum centres from 100 to 65, i.e. many centres that were contacted initially, closed down during the study period.

We received information about 1625 (73%) of those asylum seekers who had moved out into the municipalities, and 220 (93%) of those who had been referred to specialists (figure 1).

### Study end points

Among 1326 persons with negative x-rays and a Mantoux test result between 6 and 14 mm, 372 (28%) were seen in PHC, of whom 188 of 572 (33%) Africans were seen, compared to 45 of 221 (20%) Europeans and 129 of 494 (26%) Asians. Of the 568 persons with negative x-ray and Mantoux ≥ 15 mm, 86 (16%) were seen by an internist. Of all the observed proportions, the differences between Africans and Europeans seen in PHC with Mantoux 6–14 mm was the only one that was statistically significant (p < 0.05).

Further, of the 314 persons with abnormal x-ray findings, 194 ones (62%) were seen by an internist. While 165 of 235 (70%) with parenchymal findings were seen, 20 of 61 (33%) of those with other x-ray findings were seen and the differences were statistically significant (p < 0.05). Among subjects with positive x-rays, 76 of 115 (66%) Africans, 73 of 128 (57%) Asians and 38 of 55 (69%) Europeans were seen and the proportions were not significantly different.

**Figure 2:**
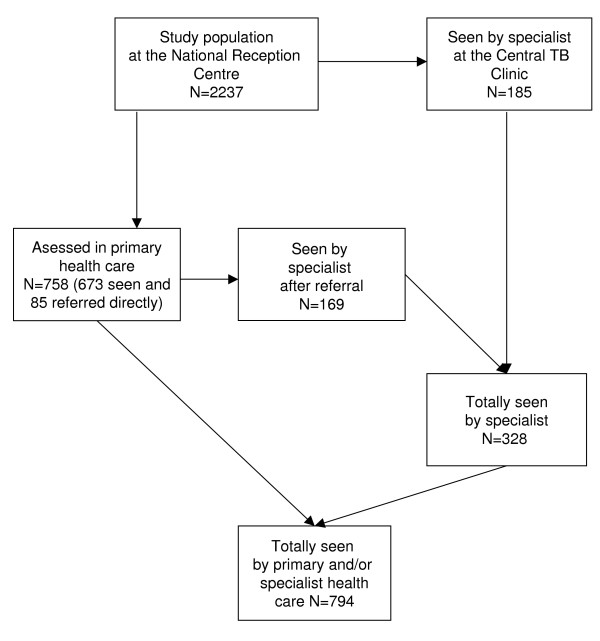
**Total numbers of asylum seekers with an indication for follow-up of TB screening seen or assessed in Norwegian primary and/or specialist health care cf. text**.

Altogether 758 were assessed in one way or another in PHC (figure 2). Of these, 673/2237 (30%) persons had actually been seen by a physician (n = 380) or nurse (n = 293) at this care level. Another 85 persons were referred directly from PHC either for chest x-ray or to a specialist without being seen personally.

Personal encounters took place a median of 9 weeks after arrival (range 0–124). Thus, 302 of 439 (69%) persons with valid dates were seen within 13 weeks of arrival and 376/439 (86%) within 26 weeks.

Three hundred and twenty-eight persons, i.e. 15% of the study group, were seen by an internist. They included 185 persons who were either examined at the Central TB Clinic and/or 169 who were examined by other hospital internists (figure 2). In all 236 persons were referred from PHC to an internist. Median time from arrival in the country to the internist visit was 25 weeks (range 0–114). The median time from the referral letter was received till the internist consultation took place was 10 weeks.

A total of 794 patients (35% of the study group) were seen either by primary and/or secondary health care providers.

When the cohort of 2237 asylum seekers were matched with the National Tuberculosis Register (31^st ^December 2006), 23 (1028/100 000) cases of active tuberculosis were identified. Thirteen were diagnosed within 3 months of arrival, 14 within 6 months, and 19 within 12 months. Of those cases, 11 were from Somalia, three from Russia and two from Serbia and Montenegro. Four cases were reported among the 27% of asylum seekers with no reply from PHC, one was seen at the Central TB Clinic, while the other three were diagnosed five, 17 and 33 weeks after arrival. We further identified 11 cases who had started treatment for latent tuberculosis, all had a Mantoux test ≥ 10 mm, and seven came from Africa.

## Discussion

Of 4643 asylum seekers, tuberculosis screening identified 2237 persons (48%) who should have been assessed and followed up by primary or specialist health care. Between 6–24 months after arrival, only one third of those at high risk had been assessed by community health care, while 15% had seen an internist. Twenty-three persons were diagnosed with tuberculosis disease and 11 had started treatment for latent tuberculosis.

### Limitations of the study

The study design implied that information about assessment and referrals depended on an initial response from PHC. The lack of response clearly reduced the number brought to our attention about asylum seekers who were referred to and seen by a specialist. Information collected through the study forms was limited to what we could retrieve from patient records. Thus, if records were not found or were incomplete, the numbers assessed and/or referred could be too low.

We do not know if the 27% with no response from PHC were assessed and followed-up to the same degree as those with a response. One possibility is, on the whole, that municipalities that did not respond were less interested in TB than those that did.

We had no access to information about asylum seekers leaving the country, and if the interval before they did so made any difference to our results. This limits our potential to give exact rates for those that should have been seen in PHC or by a specialist. Because of insufficient registration, and since the systems for handling follow-up of screening results and referrals due to symptoms are mixed together, we cannot say exactly how many of the TB cases were found through the screening system or through referrals because of symptoms. We are also unable to assess the incidence of TB at certain times after arrival. If many had left at an early stage, the denominators would decrease, with a consequent increase in rates. Dates for assessment or referral were often left open on the forms or were obviously incorrect.

Because asylum seekers do not have a personal identifier, the cases found through the match with the TB Register could have been reduced, and this may also have reduced the numbers of study individuals recorded in the municipalities and hospitals to where the study forms were sent. Apparently, this would limit both their compliance to national guidelines and the numbers found through the TB Register.

Several local authorities could not provide health records for asylum seekers who had moved on, or where asylum seeker centres had been abandoned during the study period. This also limited the quality of our study.

### Study endpoints

The entry screening was satisfactory and only 2.5% of asylum seekers had no registered Mantoux result. Voluntary screening at a similar centre in the UK reported that 94% were screened [16]. Most of the 469 asylum seekers who were not included in the total cohort left the country or were deported straight away and were of limited interest from a screening point of view. The number of the total cohort who actually got an x-ray taken, was impossible to retrieve from the data recording system at the National Reception Centre.

Everybody with a Mantoux 6–14 should have been seen in PHC, but only 28% of them were. Informal information given as reasons for this insufficient follow-up, were an absence of personal information (wrong address, name or date of birth), lack of resources, low priority, and insufficient knowledge of the guidelines or how they should be interpreted. Sometimes, patients were referred separately to x-ray, but not to a specialist. That procedure was appropriate given the previous guidelines, but not the current ones [11]. It took a median time of 9 weeks from arrival in Norway until assessment took place, but the range was 0–124 weeks. Thus, we speculate that several asylum seekers were not assessed unless they developed symptoms which brought them to the attention of the health authorities.

Follow-up of x-ray findings is the most important part of the screening, and we found that only 62% of those with initial positive findings were seen by an internist. This was most likely an organisational problem between the National Reception Centre and the Central TB Clinic. There was most probably some selection of follow up at the Central TB Clinic since relatively more cases with parenchymal findings were seen by an internist.

The guidelines recommend that all cases with Mantoux ≥ 15 mm should be referred directly to an internist, but they do not specify if the individual should be seen at the Central TB Clinic or at the nearest institution after they have moved to their new home municipality. Only 16% of those with Mantoux ≥ 15 mm were finally seen by an internist. Generally, they were not referred to the Central TB Clinic, and arguments such as an expected short stay at the National Reception Centre, the general high workload at the Central TB Clinic, and uncertainty about how long the asylum seekers would stay in the country were used to explain this. However, these subjects were not referred to a specialist after they had arrived in their new municipality, either.

The median time from the referral letter was received until the specialist consultation took place was 10 weeks. This seems appropriate if the question was an assessment for latent TB, but not if the question was to rule out active disease.

We matched study group individuals with the National Tuberculosis Register which allowed us to estimate point prevalence and an incidence rate. However, since many asylum seekers could have left the country without notification, this limited the exact population at risk. It also made it difficult for both health care levels to follow up the initial cohort. Conversely, these limitations realistically mimic the complexity of information flow and referral data for this group of individuals.

We identified 23 cases (1%) of the study group with tuberculosis disease between 6 to 24 months after arrival. Some incident cases that occurred after screening may have been mixed up with the prevalent ones, and we are unable to estimate how many cases of active TB we missed through lack of adherence to the guidelines. Studies from other countries have reported prevalences of between 0.1–1.2% [13,16-18], but valid comparisons between our and other studies require more information about the different study populations.

The number of asylum seekers who were treated for latent TB was lower than expected and potential reasons for this will be discussed in a forthcoming paper. National guidelines vary between countries, yet other studies show a lack of adherence to guidelines similar to ours [19,20]. Comparisons between studies are made even more difficult because treatment indications for latent TB vary considerably.

### Information flow and data ascertainment

An overall problem was that the flow of health information between administrative levels did not keep up with the movements of individual asylum seekers. Similar difficulties have been reported by others and compare well with our results [19-21].

Lack of adequate information between asylum seeker centres and primary or specialist health care was another problem we identified.

### Personal characteristics

Asylum seekers are registered with their name and date of birth on arrival, but do not get the personal identifier all Norwegian residents have until they have stayed in the country for several months. As a result of registration errors or because their name or date of birth are misstated, an asylum seeker may have several different identities and is not recognised in many public registers. Also, some may have entered Norway several times during the study period, resulting in a further negative influence on assessment or treatment. All these factors made follow-up quite difficult.

Asylum seekers move several times and frequently without reporting or informing authorities about their new address. They usually have little knowledge of the Norwegian language, their access to an interpreter is limited, and they may have a different conception of health issues. All are reasons why appointments were not kept.

### Consequences for policy

Despite our current and detailed guidelines, we may speculate that the inadequate follow-up was due to a complex organisation, insufficient or inappropriate information handling procedures, insufficient awareness and adherence to the guidelines, and limited focus on the TB issue by the involved health care providers and society at large. Further data analyses may cast light on some of these assumptions.

We believe that both the organisation and the guidelines should be simplified. Furthermore, a quality assurance and a report system for information flow and personal follow-up are needed. Cooperation between immigration and health authorities should make sure that municipalities with asylum seekers centres have a more streamlined and efficient system for follow-up of tuberculosis screening with special staff dedicated to implementing it.

## Conclusion

TB screening of asylum seekers identified a prevalence of 1% of active tuberculosis in the study group included for follow-up. In addition we found a considerable proportion of subjects at increased risk. We identified inadequate handling of screening results at all levels of care, with too few patients treated for latent tuberculosis.

To improve follow-up of screening results, a simplification of organisation and guidelines, introduction of quality assurance systems, and better coordination between authorities and between different levels of health care are all required.

## Abbreviations

TB: Tuberculosis; QFT: QuantiFERON^®^TB Gold; PHC: Primary Health Care.

## Competing interests

IH gave a lecture on challenges in mandatory screening of tuberculosis infection in the health care services paid by Astra Zeneca. The other authors declare that they have no competing interest.

## Authors' contributions

This paper is part of a PhD-project named "How to prevent tuberculosis among immigrants in Norway". IH has done most of the work under supervision of the other authors. GWJ was the main supervisor through the planning and conduct of the project, and through the writing process. EH contributed with input about organisational issues during the planning, implementation and writing process. SLS contributed with clinical issues during the implementation and writing process. HG contributed with public health issues during the planning, implementation and writing process. All authors have read and approved the final manuscript.

## Pre-publication history

The pre-publication history for this paper can be accessed here:


